# Compound inheritance of *EHHADH* and *MASP1* mutations contributes to nonsyndromic cleft lip: familial analysis and zebrafish models

**DOI:** 10.1242/bio.062308

**Published:** 2025-12-11

**Authors:** Paulina Swatowska, Adrian Odrzywolski, Krystian Kuźniarz, Przemko Tylzanowski

**Affiliations:** ^1^ Deptartment of Biomedical Sciences, Laboratory of Molecular Genetics, Medical University of Lublin, Lublin, 20-093, Poland; ^2^ Deptartment of Biochemistry and Molecular Biology, Medical University of Lublin, Lublin, 20-093, Poland; ^3^ Deptartment and Clinic of Maxillofacial Surgery, Medical University of Lublin, Lublin, 20-093, Poland; ^4^ Laboratory for Developmental and Stem Cell Biology, Department of Development and Regeneration, Skeletal Biology and Engineering Research Centre, University of Leuven, Leuven, 3000, Belgium

**Keywords:** Cleft lip, *EHHADH*, *MASP1*, Compound inheritance, Zebrafish, Craniofacial development, Cartilage development, Nonsyndromic

## Abstract

Cleft lip with or without cleft palate (CL/P) represents one of the most common congenital craniofacial anomalies. Its complex genetic etiology remains incompletely understood. This study investigated compound inheritance of mutations in the *EHHADH* and *MASP1* genes in a Polish family with three affected individuals using whole-genome sequencing and bioinformatic analysis, followed by zebrafish functional validation. We identified mutations in both genes that segregated with the CL/P phenotype. Network analysis demonstrated significant functional associations between these genes, with enrichment for innate immune response pathways. Using zebrafish models, we validated the phenotypic consequences of these mutations through mRNA injection experiments. Individual or combined injections of mutant *EHHADH* and *MASP1* mRNAs resulted in craniofacial abnormalities, with co-injection producing the most severe phenotypes, including cleft formation. Alcian Blue staining revealed significant alterations in cartilage development, particularly in the ceratohyal angle and chondrocyte morphology. These changes may affect extracellular matrix composition and cartilage biomechanics, potentially disrupting the structural integrity and mechanical properties essential for proper craniofacial morphogenesis. Our findings suggest the possibility of a novel genetic mechanism for nonsyndromic CL/P involving the interaction between metabolic processes regulated by *EHHADH* and immune signaling pathways controlled by *MASP1*. This study expands our understanding of the genetic complexity underlying CL/P and highlights the potential intersection of immune regulation and metabolic processes in craniofacial development.

## Teaser

**Summary:** Polish family study identifies compound inheritance of metabolic and immune gene variants that synergistically disrupt facial development, expanding our understanding of cleft lip genetics.

## INTRODUCTION

Cleft lip with or without cleft palate (CL/P) represents one of the most prevalent congenital anomalies affecting humans. This condition is characterized by incomplete fusion of hard and/or soft tissues in the oral or facial regions, resulting in significant functional and aesthetic challenges. The incidence varies significantly based on geographic location and ethnicity, with reported rates ranging from 1 to 7 cases per 1000 births (Mossey et al., 2009). The development of facial structures follows a precise timeline. Between weeks 4 and 6 of human fetal development, the primary palate forms through the fusion of the medial nasal processes and the maxillary processes. Subsequently, the secondary palate forms between weeks 6 and 10, when the palatal shelves elevate, meet at the midline, and fuse (Gritli-Linde, 2008; Starbuck et al., 2015). Disruptions during these critical periods lead to various types of cleft formations.

CL/P has been documented in over 300 syndromes, termed syndromic CL/P (SCL/P), which typically follow monogenic or chromosomal inheritance patterns (Gritli-Linde, 2008; Ludwig et al., 2016; Stuppia et al., 2011). The most common syndromic form is Van der Woude syndrome, resulting from mutations in the *IRF6* gene (locus 1q32-q41). However, nonsyndromic clefts, which represent isolated forms of this pathology, are more common.

Genetic studies have identified several key genes associated with nonsyndromic CL/P, including *IRF6*, *MAFB*, *ARHGAP29*, *VAX1*, and *PAX7*. Significant loci have been mapped to chromosomal regions 1q32, 2p13, 3q27-28, 9q21, 14q21-24, and 16q24, with population-specific variations noted between European and Asian populations (Ludwig et al., 2016; Marazita, 2012).

We report two novel mutations associated with nonsyndromic cleft lip in adjacent genes *EHHADH* and *MASP1*. *EHHADH* (Enoyl-CoA hydratase and 3-hydroxyacyl-CoA dehydrogenase), located at the 3q27.2 locus, encodes a protein that functions within peroxisomes, catalyzing reactions in the beta-oxidation pathway of fatty acids (Houten et al., 2012). While *EHHADH* is primarily recognized for its metabolic function, clinical genomic databases such as DECIPHER (Firth et al., 2009) have documented patients with *EHHADH* variants presenting with neurodevelopmental phenotypes including epileptic encephalopathy, microcephaly, and cone-rod dystrophy, suggesting potential developmental roles beyond fatty acid metabolism. The *MASP1* (MBL-associated serine protease 1) gene is located at the 3q27.3 locus and is involved in the lectin complement pathway. Mutations in *MASP1* are associated with 3MC syndrome, which causes various developmental defects, including CLP (Ashton et al., 2023; Rooryck et al., 2011). During embryonic development, *MASP1* provides guidance cues coordinating the migration of cranial neural crest cells, which contribute to craniofacial structures.

To explore the link between these mutations and the cleft phenotype, we used a zebrafish (*Danio rerio*) model organism. Zebrafish shares about 70% sequence similarity with humans and develops cleft palate abnormalities, making it a valuable model for investigating human gene function in craniofacial development (Duncan et al., 2017; Li et al., 2017).

## RESULTS

### Clinical identification of compound heterozygous variants in *EHHADH* and *MASP1*

**Fig. 1.**
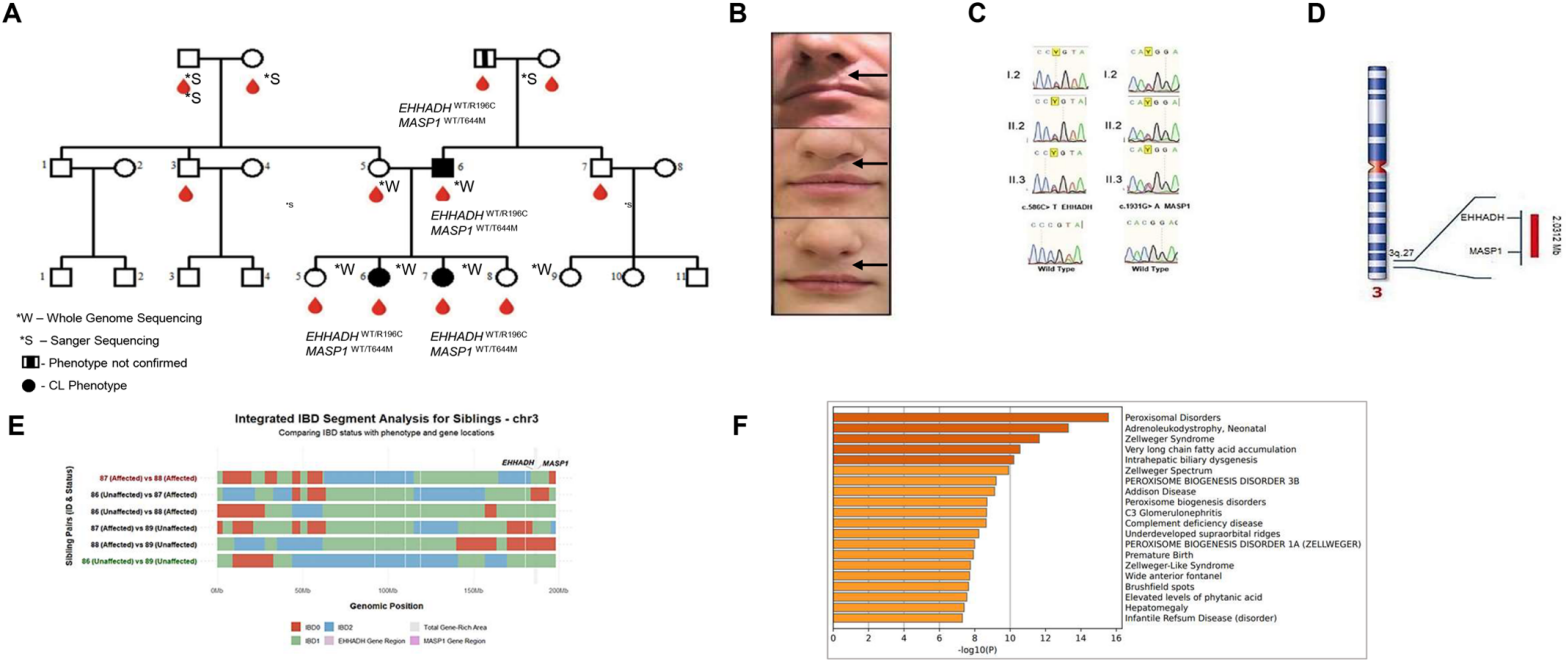
**Comprehensive genetic analysis of Polish family with nonsyndromic cleft lip.** (A) Pedigree of affected family showing inheritance pattern with blood drops indicating sample collection. Each affected family member (I.2, II.2, II.3) is heterozygous for mutations in both EHHADH and MASP1. (B) Clinical phenotypes of patients I.2, II.2, and II.3 showing cleft lip after surgical correction. Arrows indicate cleft repair sites. (C) Sanger sequencing chromatograms confirming the EHHADH c.587G>T (p. Arg196Cys) and MASP1 c.1931C>T (p. Thr644Met) variants in affected individuals compared to WT sequences. (D) Chromosomal localization showing proximity of EHHADH and MASP1 genes on chromosome 3. Created in BioRender by Gorska-janik, G. (2025). https://BioRender.com/09l01bp. CC-BY 4.0 terms. (E) Identity-by-descent (IBD) segment analysis for affected siblings on chromosome 3, demonstrating co-inheritance of variants in EHHADH and MASP1 regions using whole-genome sequencing data with stringent filtering criteria (read depth >14, genotype quality >30). (F) Disease association analysis showing significant enrichment in peroxisomal disorders (log10(*P*)=16.00) and complement deficiency diseases (log10(*P*)=−8.60), linking EHHADH and MASP 1 mutations to cleft palate through metabolic and immunological pathways (generated by MetaScape.org).

Analysis of a Polish family with three individuals affected by nonsyndromic cleft lip revealed compound heterozygous variants in two genes: *EHHADH* (c.587G>T, p. Arg196Cys) and *MASP1* (c.1931C>T, p. Thr644Met) (Fig. 1A). Whole-genome sequencing followed by comprehensive bioinformatic prioritization identified these variants as the most biologically relevant mutations associated with the observed phenotype, based on extreme population rarity (allele frequency <0.0001 for both variants), predicted deleterious functional impact, and consistent genotype-phenotype correlation across affected family members.

Population frequency analysis using gnomAD v4.1.0 revealed that the *EHHADH* variant demonstrated extreme rarity with an overall allele frequency of 4.23×10^−5^, while the *MASP1* variant exhibited an allele frequency of 2.49×10^−4^. Notably, no homozygotes were identified across the entire gnomAD dataset for either variant, suggesting potential negative selection or embryonic lethality in the homozygous state. Sanger sequencing validation (Fig. 1C) confirmed the presence of both variants in all affected family members.

The identified variants affect functionally critical domains in both proteins (Fig. S1A1,B1). The R196C mutation in *EHHADH* is located within the 3-hydroxyacyl-CoA dehydrogenase domain, essential for peroxisomal fatty acid β-oxidation, while the T644M mutation in *MASP1* resides in the peptidase S1 domain, crucial for complement cascade activation. Genomic localization analysis (Fig. 1D) revealed the physical proximity of both genes on chromosome 3, located approximately 2 Mbp apart, which explains the observed co-inheritance pattern in the affected family.

### Haplotype concordance analysis

We applied the analytical framework above to two candidate genes on chromosome 3:

**Table:** 

Gene	GRCh38 coordinates (bp)
*EHHADH*	chr3:185,190,624 - 185,281,990
*MASP1*	chr3:187,217,282 - 187,291,980

Using an interpolated recombination map for GRCh38, the corresponding genetic positions were:

*EHHADH*: 196.1223 cM

*MASP1*: 200.6316 cM.

Thus, the genetic distance between loci A and B is:

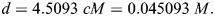
Applying the Haldane function:

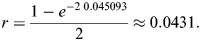
In the studied pedigree, the transmitting parent is the father, and the expected inheritance configuration is as follows: children 4 and 6 inherit haplotype 1 at both loci; children 3 and 5 inherit haplotype 2.

Substituting into Eqn (1) for *n*=4 children:

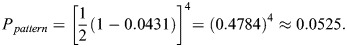
Therefore, the exact probability of observing the specified haplotype segregation pattern under the null model of Mendelian inheritance with recombination is approximately 5.25% (Fig. 1E).

### mRNA injection results support compound inheritance model

**Fig. 2.**
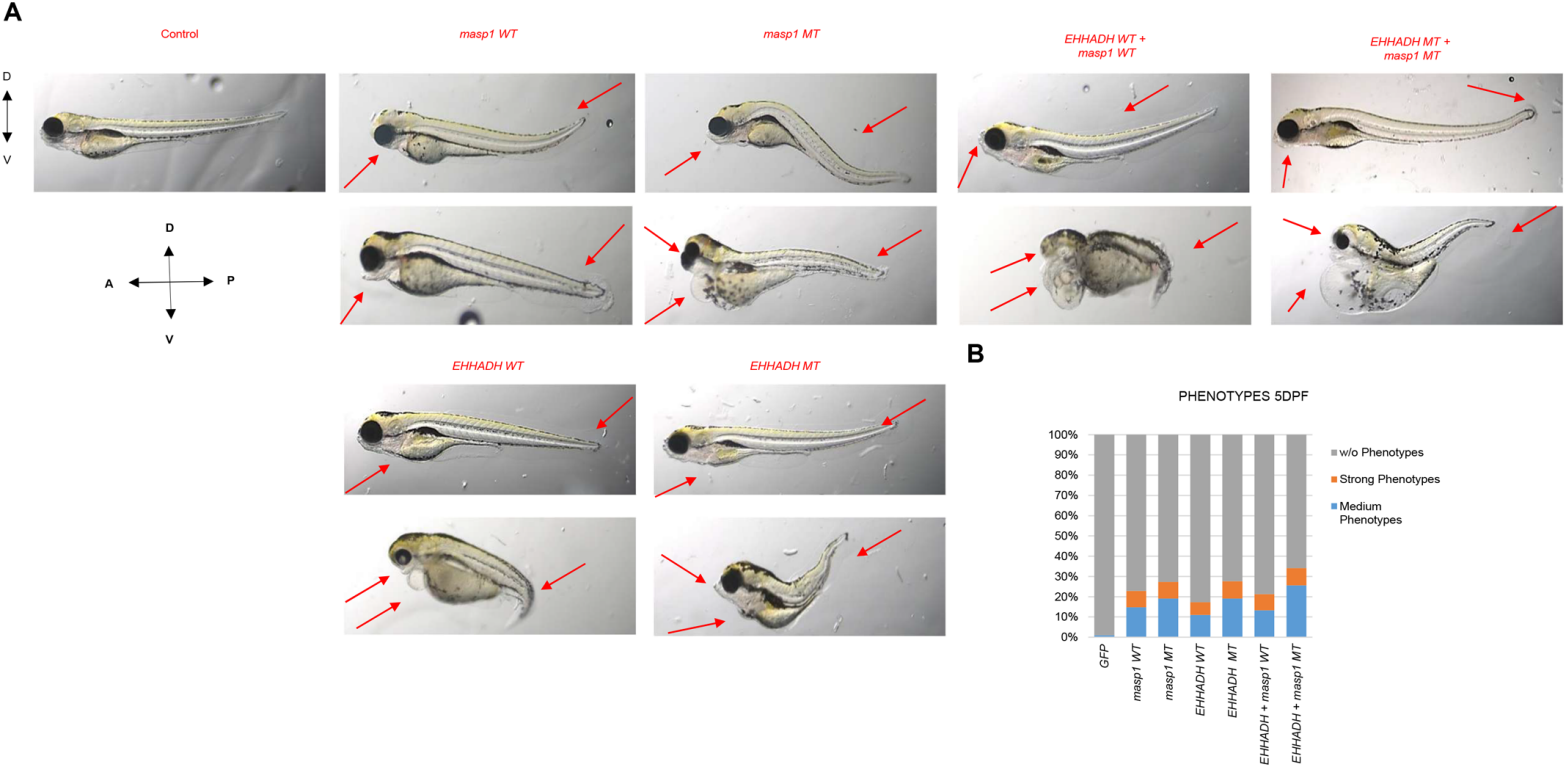
**mRNA injection phenotypes in zebrafish larvae.** (A) Representative lateral view images of 5 dpf zebrafish larvae following mRNA injection experiments (3.2× magnification). Control embryos show normal development while EHHADH and 1 variants exhibit progressive phenotypic severity including unfilled swim bladder, tail curvature, and mandibular malformations. Combined mutations demonstrate synergistic effects with severe craniofacial malformations. Directional indicators: dorsal (D), ventral (V), anterior (A), posterior (P). (B) Quantitative phenotype distribution at 5 dpf.

To validate the clinical significance of identified variants, we performed functional studies using zebrafish embryos. Microinjection of wild-type (WT) and mutant (MT) mRNA variants of *EHHADH* and *MASP1* (100 ng/μl, with GFP mRNA at 20 ng/μl as injection control) into zebrafish embryos produced developmental phenotypes that recapitulated key features of the patient presentation (Fig. 2A).

Standardized phenotype classification criteria (detailed in the Materials and Methods) were applied uniformly across all experimental groups to ensure objective assessment.

Individual *EHHADH* variants produced characteristic developmental abnormalities (Table S1). WT *EHHADH* mRNA injection resulted in 17.3% total abnormal phenotypes (6.3% strong, 11.0% medium), with 82.7% developing normally (*n*=127 larvae). MT *EHHADH* variants showed increased severity with 27.6% total abnormal phenotypes (8.5% strong, 19.1% medium; *n*=94 larvae), suggesting enhanced pathogenic activity of the MT form.

*MASP1* variants demonstrated distinct phenotypic patterns when injected alone (Table S1). WT *MASP1* injection resulted in 23% total abnormal phenotypes (8.1% strong, 14.8% medium; *n*=135 larvae), while MT *MASP1* showed 27.4% total abnormal phenotypes (8.2% strong, 19.2% medium; *n*=146 larvae), confirming increased severity of the MT variant.

The most severe abnormalities were observed upon co-injection of *EHHADH* and *MASP1* variants (Table S1). WT combination injections produced 21.3% total abnormal phenotypes (8.0% strong, 13.3% medium; *n*=150 larvae), while the MT-MT (both genes mutated) condition demonstrated additive to modest synergistic effects with 34.2% total abnormal phenotypes (8.6% strong, 25.7% medium; *n*=152 larvae). While the increase in medium phenotypes from individual MTs (∼19-20%) to combined MTs (25.7%) suggests primarily additive rather than strongly synergistic interactions, the co-injection paradigm importantly resulted in cleft-like malformations in the anterior ethmoid plate that were rarely observed with single injections alone, representing a qualitatively distinct phenotype.

Statistical analysis (Table S2) revealed significant differences between experimental groups and GFP controls (χ²=26.26, *P*<0.0001 for *EHHADH*+*MASP1* MT versus GFP). Control injections (GFP; *n*=93 larvae) resulted in 98.9% normal development with only 1.1% abnormal phenotypes, demonstrating the technical specificity of the observed effects.

We note that even WT mRNA injections produced modest phenotypic effects (17-23% abnormal), likely reflecting dosage sensitivity of these developmental regulators. Overexpression of WT *EHHADH* or *MASP1* may disrupt the finely balanced stoichiometry required during craniofacial morphogenesis. However, the observation that MT variants produce quantitatively higher abnormal rates compared to their WT counterparts (27.6% for *EHHADH* MT versus 17.3% WT; 27.4% for *MASP1* MT versus 23% WT; 34.2% for combined MT versus 21.3% combined WT) supports their pathogenic nature beyond simple dosage effects.

Statistical analysis (Table S2) confirmed significant differences between experimental groups and controls (*P*<0.0001). Medium compound phenotypes exhibited combined dorsal and ventral tail curvature and lower jaw protrusion, while strong compound phenotypes showed more severe bidirectional tail defects and extensive craniofacial malformations. These compound effects show concordance with the clinical presentation observed in the Polish family, though we acknowledge the anatomical differences between zebrafish and mammalian craniofacial development. Quantitative analysis of phenotype distribution at 5 days post-fertilization (dpf) revealed distinct patterns across experimental groups (Fig. 2B), with control injections (GFP) resulting in >99% normal development, demonstrating the specificity of the observed effects.

### Cartilage development defects confirm pathogenic mechanism

**Fig. 3.**
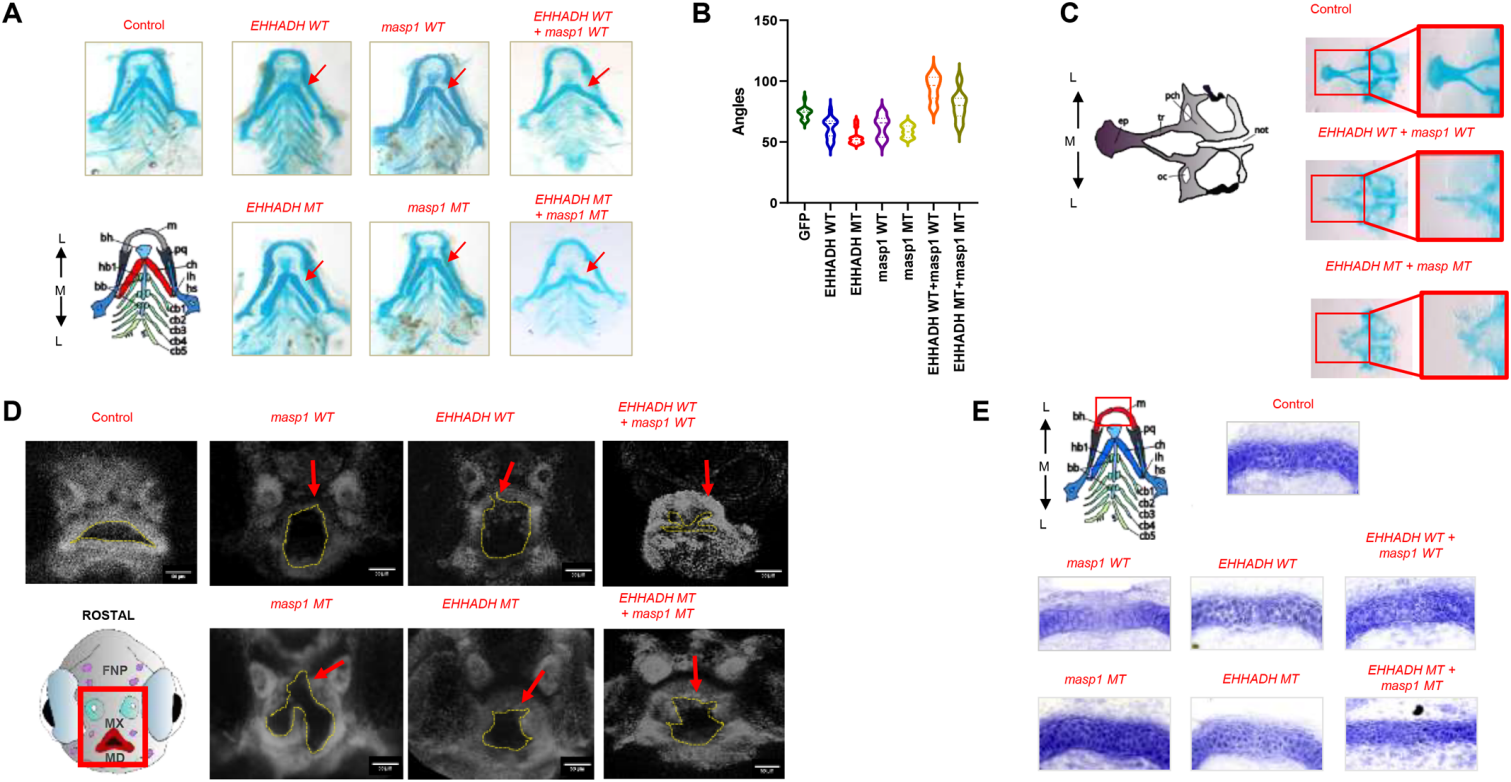
**Functional validation of *EHHADH* and *MASP1* mutations reveals craniofacial malformations in zebrafish.** (A) Alcian Blue cartilage staining (ventral view, 5 dpf). Progressive malformations observed (red arrows). Inset diagram: cartilage nomenclature (ba1-ba4, branchial arches; ch, ceratohyal; m, Meckel's cartilage; pq, palatoquadrate) with L-M orientation. (B) Ceratohyal angle quantification (*n*=20, *P*<0.0001, Kruskal–Wallis test). (C) Ethmoid plate malformations with most severe phenotype in MT combinations. Ethmoid plate anatomy (L, lateral; M, medial; D, dorsal; V, ventral; pcq, palatoquadrate process; sp, septum; fr, frontal; oc, occipital; rot, rostral process test). (D) DAPI nuclear staining (rostral view) showing cellular disruptions (red arrows). Rostral diagram: FNP (frontal nasal process), MX (maxilla), MD (mandible), M-L axis. (E) Cartilage morphology analysis showing altered cellular architecture (red boxes). (F) Detailed chondrocyte assessment revealing disrupted patterning and matrix abnormalities in experimental conditions.

To evaluate cleft formation and structural alterations in craniofacial cartilage elements, we performed Alcian Blue cartilage staining on 5 dpf zebrafish larvae using a modified Walker and Kimmel protocol (Fig. 3A). Quantitative assessment of ceratohyal cartilage angles (*n*=20 larvae per experimental group, 140 larvae total across seven conditions) using ImageJ software revealed statistically significant differences between experimental groups (*P*<0.0001, Kruskal–Wallis test) (Fig. 3B). Individual ceratohyal angle measurements for all experimental groups are provided in Table S3.

Skeletal staining analysis revealed significant cartilage defects induced by MT variants, with particular emphasis on cleft-like malformations that directly recapitulate the human patient phenotype. Notably, larvae injected with *MASP1*/*EHHADH* combination exhibited severe craniofacial malformations, with a subset developing clefts in the anterior ethmoid plate (Fig. 3C). The ethmoid plate clefts showed variable severity, ranging from minor gaps in cartilage continuity to complete separation of anterior facial structures. The most severe phenotypes occurred in embryos receiving combined *EHHADH* MT+*MASP1* MT injections, consistent with the compound inheritance model observed in the affected family.

In addition to cleft formation, comprehensive cartilage analysis revealed substantial disruptions in craniofacial cartilage development and maturation across a spectrum of severity. Quantitative assessment of ceratohyal cartilage angles was performed on *n*=20 larvae per experimental group (140 larvae total across seven conditions: GFP control, EHHADH WT, EHHADH MT, MASP1 WT, MASP1 MT, EHHADH+MASP1 WT, EHHADH+MASP1 MT), specifically selecting specimens classified as ‘normal phenotype’ to assess subtle cartilage developmental changes occurring even in morphologically normal-appearing larvae. Using ImageJ software, we measured ceratohyal angles and revealed statistically significant differences between experimental groups (*P*<0.0001, Kruskal–Wallis test) (Fig. 3B). Even larvae without overt craniofacial malformations showed significant alterations in ceratohyal angles, indicating that the mutations affect cartilage development across a spectrum of severity. These findings demonstrate that morphologically ‘normal’ larvae may harbor underlying structural defects at the cartilage level.

Analysis of chondrocyte morphology provided additional mechanistic insights into the cellular basis of these cartilage defects (Fig. 3E). Control larvae displayed normal chondrocyte organization characterized by regular cellular architecture with uniform cell shapes and sizes, intact extracellular matrix with continuous proteoglycan distribution, and organized chondrocyte columns with proper orientation along the cartilage long axis. In contrast, experimental conditions, particularly EHHADH+MASP1 MT injections, revealed multiple cellular-level defects. We observed altered chondrocyte morphology with irregular cell shapes and sizes and loss of typical rounded chondrocyte morphology, suggesting disrupted cellular differentiation. Matrix disruption was evident, with fragmented and discontinuous extracellular matrix organization and reduced Alcian Blue staining intensity indicating decreased proteoglycan content. Cellular organization defects were apparent, with loss of normal chondrocyte column formation and orientation and disorganized cellular arrangement disrupting the structural framework.

The cartilage defects ranged from subtle architectural changes such as mild disorganization of chondrocyte columns to severe structural disruptions including complete loss of cartilage continuity in cleft-like formations. We categorized this spectrum into three grades: mild alterations in ceratohyal angle with preserved overall structure, moderate defects showing visible chondrocyte disorganization and matrix irregularities, and severe phenotypes with overt cleft formation in ethmoid plate and complete structural discontinuity. These cellular-level alterations likely contribute to the structural defects observed in the craniofacial skeleton through multiple mechanisms, including disruption of extracellular matrix mechanical properties affecting tissue rigidity, impaired cell-cell and cell-matrix interactions disrupting coordinated tissue morphogenesis, and altered cartilage biomechanics compromising the structural support necessary for proper facial development. The combination of cleft formation and underlying cartilage developmental defects provides compelling evidence for the pathogenic mechanism, directly linking the molecular findings to the clinical phenotype observed in the affected family.

### Molecular mechanisms underlying variant pathogenicity

#### Structural impact on protein function

Computational analysis using UCSF ChimeraX with DynaMut2 stability prediction revealed distinct structural impacts for both mutations (Fig. S1A2, B2). The R196C mutation significantly destabilizes *EHHADH* protein structure (ΔΔG=−0.47 kcal/mol) with reduced solvent accessibility, eliminating key hydrogen bonds and disrupting electrostatic surface properties that may impair peroxisomal fatty acid oxidation pathways.

The T644M mutation in *MASP1* exhibits contrasting effects, with DynaMut2 predicting a stabilizing effect (ΔΔG=1.37 kcal/mol) while altering local interaction networks. The substitution of polar threonine with hydrophobic methionine likely modifies *MASP1*'s lectin pathway functionality, potentially disrupting complement cascade function during critical developmental windows.

#### Functional network connections reveal disease mechanism

**Fig. 4.**
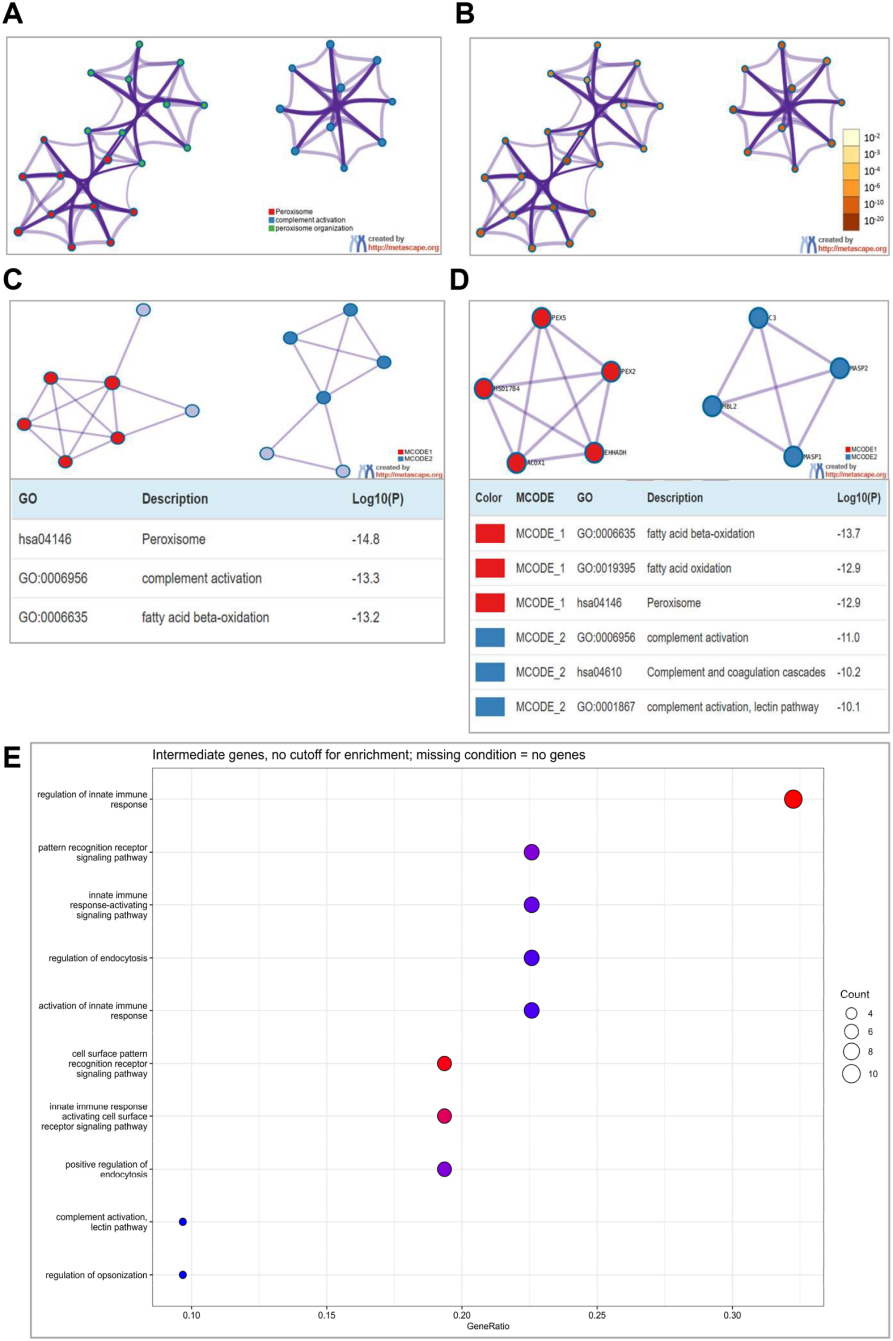
**Bioinformatic network analysis reveals functional convergence of EHHADH and MASP1 in craniofacial development.** (A,B) Functional enrichment networks showing pathway connections in cleft palate development. (C,D) Protein-protein interaction analysis with MCODE clustering revealing peroxisomal (red) and complement activation (blue) modules. (E) Statistical validation of network topology through shortest path distance analysis.

Network analysis using the GeneMania algorithm identified significant connections among *EHHADH*, *MASP1*, and other genes implicated in craniofacial development (Fig. 4A,B). The analysis revealed functionally meaningful relationships that extend beyond primary gene functions, suggesting complex biological networks underlying palatogenesis. Statistical evaluation using comparison against a null distribution revealed genes with notably low *P*-values (*P*<0.01), indicating their average shortest-path distances were significantly smaller than expected, suggesting these connections reflect biologically meaningful relationships that were previously unrecognized in the context of craniofacial development.

Protein-protein interaction analysis identified two main functional modules that provide insight into disease mechanism (Fig. S2E,D); MCODE_1 encompasses processes associated with fatty acid beta-oxidation and peroxisome function related to *EHHADH*, showing high statistical significance (LogP values of −14.8 for peroxisomes and −13.3 for complement activation) alongside high pathway scores. MCODE_2 encompasses complement activation related to *MASP1*, suggesting roles for lipid metabolism and immune response regulation during palate development. Both modules demonstrate significant interconnection, indicating that disruption of either pathway can influence craniofacial morphogenesis through distinct but converging molecular mechanisms.

#### Expression patterns during craniofacial development

We explored the mRNA expression patterns of *EHHADH* and *MASP1* during zebrafish development using single-cell RNA-seq data from Zebrahub. The spatiotemporal expression analysis revealed distinct patterns that support their involvement in craniofacial morphogenesis and provided crucial insight into the developmental windows during which these genes exert their effects.

Expression analysis during zebrafish development revealed that both *EHHADH* and *MASP1* exhibit stage-specific patterns during craniofacial morphogenesis. In neural crest tissues, both *EHHADH* and *MASP1* exhibited similar, low expression levels (∼0.02-0.03 mean expression/cell). Importantly, while mean expression values were relatively low, the proportion of cells expressing these genes differed, with *EHHADH* being expressed in a larger percentage of neural crest cells than *MASP1* (Fig. S2A,B). These tissue-specific expression patterns suggest distinct contributions to craniofacial development, with *EHHADH* showing broader tissue distribution and *MASP1* displaying more restricted expression profiles during critical developmental stages.

Temporal expression analysis revealed distinct developmental trajectories for both genes (Fig. S2C,D). Target genes exhibited peak expression at different developmental stages, with *EHHADH* showing peak expression at 5 dpf following moderate levels at earlier stages, while *MASP1* reached its highest expression at 2 dpf during early neural crest migration phases. mRNA expression patterns for both genes varied not only in intensity but also in the proportion of cells expressing each gene across developmental stages (Fig. S2D). Comparison with housekeeping genes (actb1, eef1a1l, gapdh) across all tissues and developmental stages revealed that both target genes displayed more restricted patterns, suggesting their specialized roles in specific developmental processes.

Tissue-specific expression analysis showed that *EHHADH* had its highest expression in endoderm (∼0.18), with moderate expression in intermediate mesoderm (∼0.08), neural crest tissues (∼0.02-0.03) and mesenchyme (∼0.06). Meanwhile, *MASP1* displayed moderate expression in endoderm (∼0.08-0.10), lateral mesoderm (∼0.05), and central nervous system (∼0.08).

The temporal and spatial expression patterns during key developmental periods suggest potential roles for these genes in craniofacial morphogenesis, with *MASP1* potentially functioning earlier in the developmental sequence, followed by *EHHADH* activity at slightly later stages (Fig. S2C). This temporal coordination may be critical for proper palate formation and could explain the synergistic effects observed in our functional studies.

#### Disease association analysis supports clinical relevance

Disease association analysis demonstrated significant enrichment in pathways directly relevant to the observed clinical phenotype. The analysis revealed strong connections between the studied genes and peroxisomal disorders, complement deficiency diseases, and craniofacial malformation syndromes. Top disease associations included peroxisomal disorders, adrenoleukodystrophy, neonatal adrenoleukodystrophy, Zellweger syndrome, very long chain fatty acid accumulation, Sjögren-Larsson syndrome, and multiple peroxisome biogenesis disorders (Fig. 1F).

Particularly significant were the connections to complement deficiency diseases and immunological pathways that intersect with craniofacial development through modulation of neural crest cell migration, inflammatory signaling during embryonic morphogenesis, and regulation of cellular interactions necessary for proper craniofacial morphogenesis. These findings suggest potential roles for both *EHHADH* and *MASP1* in immunological processes beyond their established roles in complement activation, indicating their involvement in craniofacial development through metabolic and immunological pathways.

The enrichment results suggest that *EHHADH* and *MASP1* mutations may disrupt neural crest cell migration by interfering with lipid metabolism pathways and immunological processes, leading to abnormal palate development and consequently to cleft formation. These findings indicate potentially novel functional relationships between these genes that may be relevant to their role in craniofacial development processes.

It is important to note that network analysis provides evidence of correlation rather than causation. While our functional studies in zebrafish support a mechanistic relationship between *EHHADH* and *MASP1*, the network connections identified through bioinformatic approaches represent predicted interactions that require further biochemical validation. Future investigations should focus on determining the precise molecular interactions between these pathways during neural crest cell migration and palatogenesis.

#### Pathogenicity assessment and variant classification

According to ACMG/AMP frequency criteria, both variants qualify as very rare (AF<0.0001) for *EHHADH* and meet supporting their potential pathogenic significance. The absence of homozygotes for both variants across >1.6 million chromosomes provide additional evidence against a benign classification, warranting detailed molecular characterization (Table 1).

**Table 1.** Comprehensive analysis of compound heterozygous variants identified in patients with cleft phenotype

Parameter	*EHHADH* c.586C>T (p.Arg196Cys)	*MASP1* c.1931C>T (p.Thr644Met)
Genomic coordinates (GRCh38)	chr3:185204740 G>A	chr3:187235940 G>A
dbSNP ID	rs202051821	rs146714674
ClinVar ID	237152	787266
Overall population frequency	4.23×10^−5^ (68/1,607,608)	2.49×10^−4^ (402/1,614,100)
European frequency	5.02×10^−5^ (59/1,175,130)	2.70×10^−4^ (319/1,180,040)
Homozygotes observed	0	0
CADD score	29.7	27.5
REVEL score	0.601	0.664
PolyPhen-2	1.00 (probably damaging)	0.998 (probably damaging)
ClinVar classification	VUS (uncertain significance)	Conflicting interpretations
ACMG/AMP criteria met	PM1+PM2+PP2+PP3	PM2+PP2+PP3
Final classification	Likely Pathogenic (LP)	VUS (tendency toward LP)
Rationale	Extreme rarity+critical domain+strong computational predictions	Moderate rarity requires clinical correlation

Classification of the *EHHADH* c.586C>T (p. Arg196Cys, rs202051821) variant according to ACMG/AMP guidelines revealed multiple lines of evidence supporting pathogenicity. The PM2 criterion was strongly met due to the variant's extreme rarity in population databases, well below the threshold for rare disease-causing variants in a functionally significant gene. The PP2 criterion was satisfied as missense variants are an established disease mechanism for *EHHADH*-related disorders, while the PM1 criterion was applicable given the variant's location within a functionally critical enzymatic domain essential for peroxisomal beta-oxidation.

The *MASP1* c.1931C>T (p. Thr644Met, rs146714674) variant presented a more complex classification scenario due to its presence across multiple populations but maintained rarity thresholds. While meeting the PM5 criterion for rarity in most populations, the significant enrichment in the Ashkenazi Jewish population required careful consideration of potential founder effects. Computational predictions (CADD=27.5, PolyPhen=0.998) indicate a high likelihood of functional impact, supporting pathogenic classification under the PP2 criterion.

Based on this analysis, the *EHHADH* R196C variant was classified as Likely Pathogenic (LP), while the *MASP1* T644M variant was classified as a Variant of Uncertain Significance (VUS) with a tendency toward LP, pending additional clinical and functional evidence.

#### Network analysis validates functional relationships

Network analysis using the GeneMania algorithm identified significant connections among *EHHADH*, *MASP1*, and other genes implicated in biological processes. Statistical evaluation using comparison against a null distribution revealed genes with notably low *P*-values (*P*<0.01), indicating their average shortest-path distances were significantly smaller than expected from random network architecture, suggesting these connections reflect biologically meaningful relationships that were previously unrecognized (Fig. 4E).

Functional enrichment analysis of intermediary genes associated with *EHHADH* and *MASP1* revealed significant overrepresentation of biological processes related to innate immune response. The lectin pathway of complement activation, particularly relevant to *MASP1*'s known biological function, and regulation of endocytosis emerged as highly enriched processes. Additional highly enriched processes included pattern recognition receptor signaling pathways, innate immune response-activating signaling pathways, and regulation of endocytosis, confirming established roles in complement activation.

The observed immunological pathways may intersect with craniofacial development through the modulation of neural crest cell migration, lipid metabolism, and inflammatory signaling during embryonic morphogenesis, where immune system components contribute to cellular interactions and structural formation of facial structures. These findings suggest a potential mechanism underlying the complex process of craniofacial development.

#### Clinical database validation confirms pathogenic significance

To further validate our findings, we examined the clinical significance of identified variants through the DECIPHER database (patient ID: 369190) (Table 2). The database analysis confirmed the presence of *EHHADH* variants in patients with complex phenotypes including abnormalities of the eye (cone/cone-rod dystrophy), musculoskeletal system (microcephaly), nervous system (epileptic encephalopathy), and other features (microcephaly, central hypotonia).

**Table 2.** DECIPHER database analysis of patient 369190 with compound heterozygous *EHHADH* variants and associated multisystem phenotype

Parameter	*EHHADH* variant 1	*EHHADH* variant 2
Patient ID	369190	369190
Genomic information		
Chromosomal location	3:185192533	3:185235444
Gene	*EHHADH*	*EHHADH*
Variant type	Sequence variant (SNV)	Sequence variant (SNV)
Genomic change	c.1577G>A	c.-92T>C
Protein change	p. Arg526His	5_prime_UTR_variant
Variant classification	missense_variant	5_prime_UTR_variant
Inheritance and pathogenicity		
Inheritance pattern	Paternally inherited	Maternally inherited
Zygosity	Heterozygous	Heterozygous
Clinical significance	Uncertain	Uncertain
Population frequencies		
gnomAD Exome MAF	6.29×10^−5^	2.74×10^−5^
gnomAD Genome MAF	8.54×10^−5^	2.63×10^−5^
Associated phenotypes		
Ocular abnormalities	Cone/cone-rod dystrophy	Cone/cone-rod dystrophy
Neurological features	Epileptic encephalopathy	Epileptic encephalopathy
Musculoskeletal features	Microcephaly	Microcephaly
Other features	Central hypotonia	Central hypotonia

Data available: https://www.deciphergenomics.org/patient/369190/phenotypes/person/318927.

Database analysis revealed that patients carrying *EHHADH* variants present with multisystem manifestations that extend beyond craniofacial features, suggesting broad developmental roles for this gene. The phenotypic associations support the multisystem effects of *EHHADH* mutations and provide additional clinical context for understanding the role of this gene in developmental disorders, including those involving craniofacial morphogenesis observed in our study.

The clinical database findings provide independent validation of *EHHADH* variant pathogenicity and demonstrate that disruption of this gene results in developmental abnormalities affecting multiple organ systems, consistent with our functional studies showing broad effects on craniofacial development. These results suggest that *EHHADH* disruption affects multiple developmental pathways, including those involved in craniofacial morphogenesis, warranting detailed clinical and functional characterization.

## DISCUSSION

This study reports the identification of potential genetic mechanisms contributing to nonsyndromic cleft lip (NCL), based on genomic analysis of a Polish family with three affected individuals. Whole-genome sequencing revealed compound heterozygous variants in two previously uncharacterized genes: *EHHADH* c.587G>T (p. Arg196Cys) and *MASP1* c.1931C>T (p. Thr644Met). While *MASP1* has been previously associated with 3MC syndrome and craniofacial malformations (Rooryck et al., 2011; Ashton et al., 2023), the present study documents the first association between *EHHADH* and craniofacial development, expanding our understanding of CL/P genetics beyond traditional developmental pathways to include peroxisomal fatty acid metabolism. The analytical estimation demonstrating a 5.25% probability of observing the specific haplotype segregation pattern under Mendelian inheritance provides statistical support for non-random co-inheritance of these variants, indicating a compound inheritance mechanism rather than classical Mendelian patterns.

Recent genome-wide association studies have identified over 40 risk loci for CL/P, yet these common variants explain only 20-25% of disease heritability (Ludwig et al., 2012; Marazita, 2012). This substantial ‘missing heritability’ problem has led researchers to investigate rare variants with larger effect sizes. Ludwig et al. (2016) demonstrated that rare coding variants contribute significantly to CL/P risk, particularly in families with multiple affected individuals (Ludwig et al., 2012). The present findings are consistent with this emerging paradigm, where oligogenic inheritance involving multiple functionally related genes may explain familial clustering patterns not captured by single-gene models.

Our compound inheritance model contrasts with both classical single-gene causes and failed metabolic gene associations. IRF6 mutations, for instance, cause Van der Woude syndrome with clear autosomal dominant inheritance and ∼96% penetrance. While these monogenic models have been extensively validated, they explain only a small fraction of CL/P cases. The metabolic gene MTHFR illustrates different challenges in establishing gene-disease associations for CL/P. Despite strong biological rationale – folate is critical for embryonic development and folate deficiency causes neural tube defects – extensive investigation of C677T and A1298C polymorphisms has yielded inconsistent results. A comprehensive 2020 meta-analysis of 31 studies (4710 NSCL/P patients, 7271 controls) found no significant association between MTHFR C677T and NSCL/P susceptibility (Imani et al., 2020).

Our findings differ in several key aspects: we observe clear genetic co-segregation in an affected family (5.25% probability under random inheritance), both variants are extremely rare (AF <0.0001 versus common MTHFR polymorphisms with AF ∼10-25%), and we demonstrate functional effects *in vivo*. Our observation that neither EHHADH nor MASP1 variants alone fully recapitulate the severe human cleft phenotype in our zebrafish model – with individual injections producing 27.6% (EHHADH MT) and 27.4% (MASP1 MT) abnormal phenotypes compared to 34.2% for combined mutations – suggests a threshold effect requiring both genetic hits. This digenic inheritance pattern, supported by the physical proximity of both genes on chromosome 3 (separated by ∼2 Mbp), may explain some of the ‘missing heritability’ in CL/P, particularly in families with multiple affected individuals where common single-gene models fail to account for observed inheritance patterns.

The extreme rarity of both variants in gnomAD v4.1.0 (*EHHADH*: AF=4.23×10^−5^; *MASP1*: AF=2.49×10^−4^) combined with the absence of homozygotes across >1.6 million chromosomes suggests' strong negative selection, potentially reflecting embryonic lethality in the homozygous state. This population genetic evidence supports the biological significance of these variants and highlights important questions about the evolutionary constraints on these genes during craniofacial development.

Recent studies have demonstrated that peroxisomal dysfunction can affect craniofacial development through disruption of lipid metabolism and cellular energy homeostasis (Steinberg et al., 2006; Wang et al., 2018; Nath et al., 2022), while *MASP1* has been established as a key regulator of neural crest cell migration in multiple model systems.

Functional validation using zebrafish embryos provided compelling evidence for compound effects between *EHHADH* and *MASP1* mutations. The observation that combined MT injections produced 34.2% abnormal phenotypes compared to 27.6% for *EHHADH* alone and 27.4% for *MASP1* alone demonstrates clear additive effects with qualitatively distinct outcomes. While the increase in medium phenotypes from individual MTs (∼19-20%) to combined MTs (25.7%) suggests primarily additive rather than strongly synergistic interactions, the co-injection paradigm importantly resulted in cleft-like malformations in the anterior ethmoid plate that were rarely observed with single injections alone, representing a qualitatively distinct phenotype. Most importantly, the identification of clefts in the anterior ethmoid plate following co-injection of MT variants provides the first functional evidence linking these genes to cleft formation . The significant alterations in ceratohyal cartilage angles (*P*<0.0001) and chondrocyte morphology disruptions suggest that these variants affect fundamental cartilage development processes, likely through alterations in extracellular matrix composition and cellular organization.

Single-cell RNA sequencing analysis revealed distinct spatiotemporal expression patterns consistent with a sequential activation model during craniofacial morphogenesis. *MASP1* peaked at 2 dpf during early neural crest migration phases, while *EHHADH* reached maximum expression at 5 dpf during cartilage maturation. This temporal coordination indicates complementary developmental functions with *MASP1* regulating early patterning events and *EHHADH* contributing to structural remodeling processes. These findings support the hypothesis that disruption of this temporal cascade could lead to cumulative developmental defects, with early *MASP1*-mediated immune signaling abnormalities compounded by later *EHHADH*-related metabolic disruptions.

Bioinformatic analysis revealed unexpected functional convergence between peroxisomal fatty acid metabolism and innate immune response pathways. Network analysis identified statistically significant connections (*P*<0.01) between *EHHADH* and *MASP1*, with protein-protein interaction analysis revealing two main functional modules: MCODE_1 centered on peroxisomal β-oxidation (Log*P*=−14.8) and MCODE_2 associated with complement activation (Log*P*=−13.3). This finding indicates previously unrecognized crosstalk between metabolic and immunological processes during craniofacial morphogenesis, which is consistent with emerging evidence for metabolic-immune interactions in developmental biology.

Disease association analysis demonstrated significant enrichment in peroxisomal disorders (log₁₀(*P*)=−16.00) and complement deficiency diseases (log₁₀(*P*)=−8.60), supporting the biological relevance of these pathways to craniofacial development. Clinical validation from the DECIPHER database identified patient (id: 369190) carrying compound heterozygous *EHHADH* variants and presenting with multisystem developmental abnormalities, including cone/cone-rod dystrophy, epileptic encephalopathy, and microcephaly, thus providing independent clinical evidence for broader developmental roles of *EHHADH* extending beyond craniofacial morphogenesis.

The identification of *EHHADH* as a candidate gene reveals previously unrecognized links between peroxisomal metabolism and craniofacial development. Peroxisomal disorders have been associated with craniofacial dysmorphisms, however, the mechanisms remain poorly understood (Carmichael and Schrader, 2022; Yang et al., 2023)^.^The present structural analysis revealing that the R196C mutation significantly destabilizes *EHHADH* protein structure (ΔΔG=−0.47 kcal/mol) while the T644M mutation paradoxically stabilizes *MASP1* (ΔΔG=1.37 kcal/mol) but alters local interaction networks suggests that these variants may disrupt cellular processes through different molecular mechanisms. We propose that *EHHADH* dysfunction may affect neural crest cell migration through alterations in membrane lipid composition, energy metabolism, or lipid signaling pathways, while *MASP1* alterations may disrupt complement-mediated cellular interactions during critical developmental windows.

Carmona-Fontaine et al. demonstrated that complement proteins regulate neural crest cell migration through direct effects on cellular adhesion and motility (Carmona-Fontaine et al., 2011). The present findings suggest that metabolic processes may similarly influence neural crest behavior, potentially through effects on membrane composition or energy metabolism during migration. This observation supports the hypothesis that both complement signaling and metabolic regulation contribute to proper craniofacial development through a convergent pathway involving extracellular matrix remodeling and cellular energy homeostasis.

We acknowledge important limitations of our study. Zebrafish lack a secondary palate, and the observed ethmoid plate clefts represent analogous rather than homologous structures to human cleft lip. Our findings demonstrate that these variants disrupt fundamental craniofacial morphogenesis processes rather than specifically recapitulating human CL/P anatomy. Additionally, our mRNA overexpression approach, while providing functional validation, does not fully recapitulate the heterozygous state in patients. We are developing CRISPR MT lines with the specific point mutations, though this has proven technically challenging. Network analysis provides evidence of correlation rather than direct causation, requiring further biochemical validation.

Based on these findings, we propose a novel compound inheritance model for nonsyndromic cleft lip involving the interaction between metabolic processes regulated by *EHHADH* and immune signaling pathways controlled by *MASP1*. This model proposes that neither variant alone is sufficient to cause disease, but their combination creates a threshold effect that disrupts the delicate balance of cellular processes required for proper palatogenesis. Future investigations should determine whether this compound inheritance pattern extends to other metabolic-immune gene pairs and whether similar mechanisms contribute to other craniofacial malformations.

While this study represents a focused analysis of a single family, which is methodologically appropriate for identifying novel genetic mechanisms, we performed comprehensive database analyses including DECIPHER clinical genomics data and gnomAD population frequencies, which provided independent validation of variant pathogenicity and broader context for our findings. The mechanistic connections between metabolic and immune pathways in craniofacial development, although supported by network analysis and functional studies, warrant further biochemical characterization to fully elucidate the molecular mechanisms underlying this compound inheritance pattern. Future investigations should focus on determining the precise cellular and molecular interactions between these pathways during neural crest cell migration and palatogenesis.

## MATERIALS AND METHODS

### Ethical approval

The study protocol was approved by the institutional Bioethics Committee (reference number: KE-0254/130/2021). The study was conducted in full compliance with relevant ethical guidelines and regulations. All participants provided written informed consent prior to their involvement in the research procedures.

### Patients and DNA isolation

The study cohort consisted of a Polish family with three individuals affected by nonsyndromic cleft lip: the proband, her sister, and their father. Additionally, we recruited two unaffected sisters and the unaffected mother of the proband, who were supervised by the Clinic of Maxillofacial Surgery of the Medical University of Lublin, Poland (Fig. 1A,B). Genomic DNA from all family members (affected and unaffected) was isolated from peripheral blood samples using the MiniBlood Kit (A&A Biotechnology, Gdynia, Poland) following the manufacturer's recommendations.

### Whole-genome sequencing and bioinformatic analysis

Whole-genome sequencing (WGS) was performed at the Genomics Core facility (UZ – KU Leuven, Belgium). The raw data were processed according to the Broad Institute's Best Practices pipeline (McKenna et al., 2010). The files were received in variant calling format (VCF), containing all SNPs and small INDELs from all analyzed patients. Variant quality was assessed and filtered using Variant Quality Score Recalibration. Variants were subsequently annotated using the Ensembl Variant Effect Predictor (VEP) (McLaren et al., 2016) with cache version 101. The results of WGS in the form of VCF files were subjected to two different bioinformatics analyzes using distinctive algorithms and databases. The Exomiser (Robinson et al., 2014), using applied filtering, provided many candidate variants for CLP.

### gnomAD Population frequency analysis

Population frequency analysis of the identified variants was performed using the Genome Aggregation Database (gnomAD) version 4.1.0 (Karczewski et al., 2020), which aggregates high-quality exome and genome sequencing data from 1,614,100 chromosomes across diverse populations. This analysis is crucial for variant interpretation according to ACMG/AMP guidelines, as allele frequency is a key criterion for pathogenicity assessment.

For each variant, we extracted the following parameters from gnomAD: overall allele frequency (AF), population-specific frequencies across major ancestry groups (African/African American, Latino/Admixed American, Ashkenazi Jewish, East Asian, Finnish, Non-Finnish European, South Asian), allele count (AC), allele number (AN), and homozygote count. This data was used to assess variant rarity and population distribution patterns, which are essential for clinical interpretation.

Variants were classified according to ACMG/AMP frequency thresholds: very rare (AF<0.0001), rare (AF 0.0001-0.001), and common (AF>0.001). Population heterogeneity was assessed by comparing frequencies across different ancestry groups to identify potential founder effects or population-specific enrichment patterns (Richards et al., 2015).

### Clinical genomic database analysis

To assess the clinical relevance of identified variants, we queried the DECIPHER database for documented cases with *EHHADH* and *MASP1* variants (Firth et al., 2009). DECIPHER aggregates genomic variants and associated phenotypes from over 25,000 patients worldwide. We extracted information on variant types, inheritance patterns, and clinical phenotypes associated with variants in our genes of interest. This analysis was performed to identify potential genotype-phenotype correlations and assess whether our identified variants were consistent with previously reported clinical presentations.

### Analytical estimation of haplotype concordance probability

We consider a nuclear family with *n* offspring and phased parental haplotypes. A putative pathogenic variant is assumed to reside on a specific haplotype inherited from one parent (the transmitting parent). Our objective is to analytically compute the probability that a specific subset of children containing k children, inherits the same disease-linked haplotype across two loci, denoted as A and B.

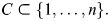
This configuration is only possible if each child in *C* inherits the same haplotype (haplotype 1) from the transmitting parent at both loci A and B, and each child in C′ (i.e. the children not in C) inherits the alternate haplotype (haplotype 2), without a recombination event occurring between loci A and B.

Let loci A and B lie on the same chromosome, separated by genetic distance d measured in centiMorgans (cM). Under the assumption of no crossover interference, the Haldane mapping function provides the recombination fraction *r*∈[0, 0.5] as:

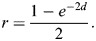
This reflects the probability that a recombination event occurs between loci A and B in a single meiosis.

In the absence of recombination, each child inherits one of the two unbroken haplotypes from the transmitting parent with equal probability. The probability that a child receives a specific haplotype (e.g. haplotype 1) without recombination is:

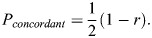
This probability applies symmetrically to both expected inheritance scenarios (haplotype 1 or 2).

Assuming independence of meiotic segregation across offspring, the probability that all *n* children receive the expected haplotypes – i.e. all in C inherit haplotype 1 and all in inherit haplotype 2 – is:

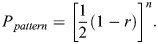
This expression gives the exact probability of observing a specific bipartition of haplotype inheritance across two loci, under Mendelian segregation and given recombination.

### Identity-by-descent estimation

To determine the haplotype configurations at the loci of interest, identity-by-descent (IBD) segments were identified using the approach described by Odrzywolski et al. (Odrzywolski et al., 2024). Briefly, IBD detection was performed from whole-genome sequencing data using a multi-sample VCF, with stringent filters: read depth >14, genotype quality >30, no Mendelian error, and minimal allelic imbalance. For each pair of affected individuals, allele depth ratios were compared at all informative SNPs to detect consistent inheritance from a shared parental haplotype. IBD regions were defined as continuous genomic intervals in which at least one allele was inherited identically by descent (IBD1 or IBD2) (Fig. 1E).

### Computational analysis of structural and functional consequences of the R196C mutation in *EHHADH* and T644M mutation in *MASP1*

The structural and functional consequences of two distinct mutations – R196C in the peroxisomal bifunctional enzyme *EHHADH* and T644M in mannan-binding lectin serine peptidase 1 (*MASP1*) – were investigated through an integrated computational approach. The WT protein structures were obtained from the AlphaFold database (AF-Q08426-F1-model_v4.pdb for *EHHADH* and P487402_b57b3 for *MASP1*), and the mutation was introduced using UCSF ChimeraX with rotamer optimization based on the Dunbrack library (Jumper et al., 2021; Pettersen et al., 2021; Shapovalov and Dunbrack, 2011). Structural alignment between WT and MT proteins was performed using ChimeraX's matchmaker tool with the Needleman-Wunsch algorithm and BLOSUM-62 matrix. Protein stability changes were predicted using DynaMut2, which calculated the free energy change (ΔΔG) associated with the mutation (Rodrigues et al., 2021). Solvent-accessible surface area (SASA) was analyzed for both variants to evaluate changes in residue exposure (Miller et al., 1987). Intermolecular interactions were characterized through hydrogen bond analysis and contact mapping within a 5Å radius of the mutation site (McDonald and Thornton, 1994). Electrostatic potential surfaces were generated to assess alterations in charge distribution (Miller et al., 1987). The functional implications of the R196C mutation in *EHHADH* were evaluated with respect to peroxisomal fatty acid oxidation pathways, while the consequences of the T644M mutation in *MASP1* were assessed in the context of complement activation and inflammatory response pathways. Correlations between structural disruptions and known biological functions were established to predict the potential physiological impact of each mutation on their respective molecular systems.

### Bioinformatic analysis of *EHHADH* and *MASP1* mutations in non-syndromic cleft palate pathogenesis

To identify the molecular mechanisms underlying non-syndromic cleft palate, we conducted a comprehensive bioinformatic analysis of mutations identified in *EHHADH* and *MASP1* genes from affected patients. We employed a multi-level analytical approach using Metascape.org platform encompassing (Zhou et al., 2019): analysis of enriched term networks using functional clustering algorithms to determine connections between biological processes involved in palate development; protein-protein interaction (PPI) analysis utilizing the MCODE algorithm to identify functionally related protein modules (Bader and Hogue, 2003); and disease association studies using the DisGeNET database to establish connections between identified genes and specific disease entities (Piñero et al., 2020). All statistical analyses were performed using *P*-values adjusted by the Benjamini-Hochberg method, with a statistical significance threshold of *P*<0.05. For assessment of association strength, logarithmic transformation (log_10_(p)) was applied (Benjaminit and Hochberg, 1995). Results were presented as interaction networks and bar graphs showing the statistical significance of identified associations.

### Evaluation of gene network associations through randomization analysis

To assess the statistical significance of *EHHADH*-*MASP1* network connections, we employed network randomization with a GeneMANIA-based null model to determine whether the observed shortest path distances differed significantly from those expected by chance (Warde-Farley et al., 2010). The null model preserved the original network's degree distribution and edge weight distribution while randomizing connections. We performed 1000 simulations in batches of 200 for computational efficiency, generating a distribution of average shortest-path distances for each node. For each gene, we compared the observed average shortest-path distance against the null distribution generated through simulations. The raw *P*-value was calculated as the proportion of simulations where the simulated distance was less than or equal to the observed distance.

Significant genes identified from this network analysis were subsequently analyzed for functional enrichment using the clusterProfiler package in R (Yu, 2024; R: The R Project for Statistical Computing). We performed Gene Ontology (GO) term enrichment analysis to identify biological processes, molecular functions, and cellular components associated with these genes, providing further biological context to our findings.

### Methods for single-cell RNA sequencing data analysis of *EHHADH* and *MASP1* in zebrafish

Single-cell RNA sequencing (scRNA-seq) data from the Zebrafish Hub atlas was analyzed to investigate the expression patterns of *EHHADH* and *MASP1* genes. The dataset contained gene expression measurements across multiple developmental stages and anatomical structures of zebrafish (*D. rerio*).

All analyses were performed using R-based packages. The data was imported from H5AD format using the zellkonverter package (GitHub - theislab/zellkonverter: Conversion between scRNA-seq objects).

Expression patterns of *EHHADH* and *MASP1* were extracted from the dataset and analyzed across different developmental stages and anatomical structures. Housekeeping genes (*actb1*, *eef1a1l1*, and *gapdh*) were included as reference controls to provide context for the expression levels of our genes of interest.

For each gene, we calculated several metrics, including mean expression level in each developmental stage and anatomical structure, percentage of cells expressing each gene (defined as expression value >0), and cell count for each stage and anatomical structure.

We performed two parallel analyses. In a developmental stage analysis, we examined gene expression across multiple zebrafish developmental timepoints, ranging from early embryonic stages through larval development. This allowed us to identify temporal changes in expression of *EHHADH* and *MASP1*.

In anatomical structure analysis, we investigated gene expression across different tissues and anatomical structures to characterize spatial expression patterns. This analysis helped us determine the tissues with the highest expression of our target genes. Only developmental stages and anatomical structures with at least 30 cells were included in the final analysis.

Expression patterns were visualized using dot plots and bar charts. In the dot plots, dot size represents the percentage of cells expressing a gene, while color intensity represents the mean expression level. Bar charts directly compare the mean expression levels of *EHHADH* and *MASP1* across developmental stages and anatomical structures. For analysis purposes, genes were categorized as either target genes (*EHHADH* and *MASP1*) or housekeeping genes for comparison. The combined expression level of target genes ordered anatomical structures.

### Generation of *EHHADH* and *MASP1* full-length mRNA

The coding sequence of *EHHADH* was amplified by PCR from cDNA derived from HEK293 cells using the following primers: *EHHADH*_F (5′-ATGGCCGAGTATACGC −3′) and *EHHADH*_R (5'-TCACAATTTACTGCTAGGGGA −3′). The coding sequence of *MASP1* was amplified by PCR from cDNA derived from zebrafish embryos using primers:

*MASP1*_F (5′-ATGGAGCTTACACGTGTCTTTGTGATC −3′)

*MASP1*_R (5′-TCACCACCAGCGCTCCG −3′).

PCR conditions were as follows: hot start at 98°C for 30 s, followed by 35 cycles of 98°C for 10 s, 62°C for 30 s, and 72°C for 2 min, with a final extension at 72°C for 5 min using Q5 High-Fidelity DNA Polymerase (New England Biolabs, Ipswich, MA, USA).

The amplified products were cloned into the pCS2+ vector using Gibson Assembly (New England Biolabs) to generate pCS2+_ *EHHADH* WT and pCS2+_*MASP1* WT. The sequence of the genes was confirmed by Sanger sequencing.

To create MT mRNA, the Q5 Site-Directed Mutagenesis Kit (New England Biolabs) was used with plasmids pCS2+_ *EHHADH* WT and pCS2+_*MASP1* WT as templates. The following primers were used: EHH_SDM_F (5′-CTAGAATCCTGTAGACTCTGC −3′), EHH_SDM_R (5′-CTCTCAAAGTCTAGTTGGA −3′), *MASP1*_SDM_F (5′-CTATAATATCAtgGGTAATATGTTCTGCGCT-3′), and *MASP1*_SDM_R (5′-TTAACAGATCTCGACGCATA −3′). To generate *EHHADH* and *MASP1* mRNA, pCS2+ was linearized with NsiI and transcribed using the Sp6 mMessage Machine kit (Thermo Fisher Scientific, Waltham, MA, USA).

### Zebrafish embryo manipulation and skeletal analysis

WT zebrafish (*D. rerio*, AB strain) were maintained according to standard protocols at 28.5°C on a 14-h light/10-h dark cycle. Animal procedures were performed in accordance with protocols approved by the institutional Animal Care and Use Committee.

Synthesized mRNA was microinjected into zebrafish embryos at the single-cell stage. Each embryo received approximately 1nl of mRNA solution (concentration range: 30-300 ng/μl, optimized to 100 ng/μl based on phenotypic consistency) introduced into the yolk using a WPI 3301R micromanipulator (World Precision Instruments, Sarasota, FL, USA) and IM-300 pneumatic microinjector (Narishige, Precoptic, Warsaw, Poland). GFP mRNA (20 ng/μl) served as an injection control. Following injection, embryos were kept in E3 medium at 28.5°C and scored for survival and phenotypic characteristics.

Phenotypic assessment was performed daily from 1 to 5 dpf using a ZEISS SteREO Discovery.V8 stereomicroscope (Carl Zeiss AG, Oberkochen, Germany). Developmental abnormalities were documented and categorized based on observed phenotypes. Larvae were fixed in 4% paraformaldehyde at 5 dpf for subsequent skeletal analysis.

Cartilage staining was performed using an acid-free Alcian Blue protocol as described by Walker and Kimmel (2007). Imaging was performed using a ZEISS SteREO Discovery.V8 stereomicroscope, and ceratohyal angles were measured using ImageJ software (version 1.53c, NIH, USA). Statistical analysis of phenotypic data and morphometric measurements was performed using GraphPad Prism software (version 8.4.3, GraphPad Software, San Diego, CA, USA). A *P*-value <0.05 was considered statistically significant.

### Phenotype classification criteria

Standardized classification criteria were applied uniformly across all experimental conditions. Normal phenotypes were defined as larvae with no observable developmental defects, including normal body axis, complete swim bladder inflation at 5 dpf, and normal craniofacial morphology without asymmetry or malformations. Medium phenotypes presented with one or more moderate developmental abnormalities such as non-inflated or partially inflated swim bladder, mild-to-moderate tail curvature (ventral or dorsal), mandibular protrusion or mild jaw asymmetry without severe dysmorphology, or minor craniofacial asymmetry. Strong phenotypes displayed two or more severe developmental features including cardiac edema, severe bidirectional tail malformations (both dorsal and ventral curvature), pronounced craniofacial disruptions including jaw malformation, cleft-like defects in facial structures, or severe overall body axis defects.

### DAPI nuclear staining for craniofacial malformation assessment

To visualize craniofacial malformations at the cellular level, zebrafish larvae were processed for DAPI nuclear staining at 5 dpf. Following phenotypic assessment, specimens were briefly washed with 0.05% PBST and incubated with DAPI (Thermo Fisher Scientific) diluted 1:1000 in 0.05% PBST for 30 min at room temperature. Samples were subsequently washed three times for 20 min each in 0.05% PBST and stored in PBS at 4°C until imaging (Maili et al., 2023).

For rostral-view morphological analysis, larvae were mounted in Costar culture dishes containing 2% low-melt agarose. Each specimen was oriented face-forward by manipulating the tail position to ensure consistent frontal presentation with eyes positioned on the same horizontal plane. DAPI fluorescence was visualized using confocal microscopy to assess nuclear organization and identify structural abnormalities within craniofacial tissues. This complementary approach provided detailed visualization of cellular-level malformations associated with experimental conditions.

### Ethics statement

Human study procedures were approved by the institutional Bioethics Committee (KE-0254/130/2021). Written informed consent for participation and publication of clinical data and images was obtained from all participants.

## Supplementary Material

10.1242/biolopen.062308_sup1 Supplementary information

## References

[BIO062308C1] Ashton, C. J., Perveen, R., Beaman, G., Crisponi, G., González-Del Angel, A., Garza-Mayén, G., Alcántara-Ortigoza, M. A., O'Sullivan, J. and Clayton-Smith, J. (2023). 3MC syndrome: Molecular findings in previously reported and milder patients expand the natural history and phenotypic spectrum. *Clin. Dysmorphol.* 32, 7-13. 10.1097/MCD.0000000000000443 36503917

[BIO062308C2] Bader, G. D. and Hogue, C. W. V. (2003). An automated method for finding molecular complexes in large protein interaction networks. *BMC Bioinformatics* 4, 1-27. 10.1186/1471-2105-4-2 12525261 PMC149346

[BIO062308C3] Benjaminit, Y. and Hochberg, Y. (1995). Controlling the false discovery rate: a practical and powerful approach to multiple testing. *J. R. Stat. Soc. Series B Stat. Methodol.* 57, 289-300. 10.1111/j.2517-6161.1995.tb02031.x

[BIO062308C4] Carmichael, R. E. and Schrader, M. (2022). Determinants of peroxisome membrane dynamics. *Front. Physiol.* 13, 834411. 10.3389/fphys.2022.834411 35185625 PMC8853631

[BIO062308C5] Carmona-Fontaine, C., Theveneau, E., Tzekou, A., Tada, M., Woods, M., Page, K. M., Parsons, M., Lambris, J. D. and Mayor, R. (2011). Complement fragment C3a controls mutual cell attraction during collective cell migration. *Dev. Cell* 21, 1026-1037. 10.1016/j.devcel.2011.10.012 22118769 PMC3272547

[BIO062308C6] Duncan, K. M., Mukherjee, K., Cornell, R. A. and Liao, E. C. (2017). Zebrafish models of orofacial clefts. *Dev. Dyn.* 246, 897-914. 10.1002/dvdy.24566 28795449 PMC5777297

[BIO062308C7] Firth, H. V., Richards, S. M., Bevan, A. P., Clayton, S., Corpas, M., Rajan, D., Vooren, S. V., Moreau, Y., Pettett, R. M. and Carter, N. P. (2009). DECIPHER: database of chromosomal imbalance and phenotype in humans using ensembl resources. *Am. J. Hum. Genet.* 84, 524-533. 10.1016/j.ajhg.2009.03.010 19344873 PMC2667985

[BIO062308C8] **GitHub repository** theislab/zellkonverter: Conversion between scRNA-seq objects. Available at: https://github.com/theislab/zellkonverter

[BIO062308C9] Gritli-Linde, A. (2008). Chapter 2 the etiopathogenesis of cleft lip and cleft palate. Usefulness and caveats of mouse models. *Curr. Top. Dev. Biol.* 84, 37-138. 10.1016/S0070-2153(08)00602-9 19186243

[BIO062308C10] Houten, S. M., Denis, S., Argmann, C. A., Jia, Y., Ferdinandusse, S., Reddy, J. K. and Wanders, R. J. A. (2012). Peroxisomal L-bifunctional enzyme (Ehhadh) is essential for the production of medium-chain dicarboxylic acids. *J. Lipid Res.* 53, 1296-1303. 10.1194/jlr.M024463 22534643 PMC3371241

[BIO062308C11] Imani, M. M., Golchin, N., Safaei, M., Rezaei, F., Abbasi, H., Sadeghi, M., Lopez-Jornet, P., Mozaffari, H. R. and Sharifi, R. (2020). Methylenetetrahydrofolate reductase C677T polymorphism is not associated with the risk of nonsyndromic cleft lip/palate: An updated meta-analysis. *Sci. Rep.* 10, 1531. 10.1038/s41598-020-58357-0 32001764 PMC6992667

[BIO062308C12] Jumper, J., Evans, R., Pritzel, A., Green, T., Figurnov, M., Ronneberger, O., Tunyasuvunakool, K., Bates, R., Žídek, A., Potapenko, A. et al. (2021). Highly accurate protein structure prediction with AlphaFold. *Nature* 596, 583-589. 10.1038/s41586-021-03819-2 34265844 PMC8371605

[BIO062308C13] Karczewski, K. J., Francioli, L. C., Tiao, G., Cummings, B. B., Alföldi, J., Wang, Q., Collins, R. L., Laricchia, K. M., Ganna, A., Birnbaum, D. P. et al. (2020). The mutational constraint spectrum quantified from variation in 141,456 humans. *Nature* 581, 434-443. 10.1038/s41586-020-2308-7 32461654 PMC7334197

[BIO062308C14] Li, E. B., Truong, D., Hallett, S. A., Mukherjee, K., Schutte, B. C. and Liao, E. C. (2017). Rapid functional analysis of computationally complex rare human IRF6 gene variants using a novel zebrafish model. *PLoS Genet.* 13, e1007009. 10.1371/journal.pgen.1007009 28945736 PMC5628943

[BIO062308C15] Ludwig, K. U., Mangold, E., Herms, S., Nowak, S., Reutter, H., Paul, A., Becker, J., Herberz, R., AlChawa, T., Nasser, E. et al. (2012). Genome-wide meta-analyses of nonsyndromic cleft lip with or without cleft palate identify six new risk loci. *Nat. Genet.* 44, 968-971. 10.1038/ng.2360 22863734 PMC3598617

[BIO062308C16] Ludwig, K. U., Ahmed, S. T., Böhmer, A. C., Sangani, N. B., Varghese, S., Klamt, J., Schuenke, H., Gültepe, P., Hofmann, A., Rubini, M. et al. (2016). Meta-analysis reveals genome-wide significance at 15q13 for nonsyndromic clefting of both the lip and the palate, and functional analyses implicate GREM1 as a plausible causative Gene. *PLoS Genet.* 12, e1005914. 10.1371/journal.pgen.1005914 26968009 PMC4788144

[BIO062308C17] Maili, L., Ruiz, O. E., Kahan, P. H., Chiu, F., Larson, S. T., Hashmi, S. S., Hecht, J. T. and Eisenhoffer, G. T. (2023). Facial analytics based on a coordinate extrapolation system (zFACE) for morphometric phenotyping of developing zebrafish. *Dis. Model. Mech.* 16, dmm049868. 10.1242/dmm.049868 37102214 PMC10245138

[BIO062308C18] Marazita, M. L. (2012). The evolution of human genetic studies of cleft lip and cleft palate. *Annu. Rev. Genomics Hum. Genet.* 13, 263-283. 10.1146/annurev-genom-090711-163729 22703175 PMC3760163

[BIO062308C19] McDonald, I. K. and Thornton, J. M. (1994). Satisfying hydrogen bonding potential in proteins. *J. Mol. Biol.* 238, 777-793. 10.1006/jmbi.1994.1334 8182748

[BIO062308C20] McKenna, A., Hanna, M., Banks, E., Sivachenko, A., Cibulskis, K., Kernytsky, A., Garimella, K., Altshuler, D., Gabriel, S., Daly, M. et al. (2010). The genome analysis toolkit: a MapReduce framework for analyzing next-generation DNA sequencing data. *Genome Res.* 20, 1297-1303. 10.1101/gr.107524.110 20644199 PMC2928508

[BIO062308C21] McLaren, W., Gil, L., Hunt, S. E., Riat, H. S., Ritchie, G. R. S., Thormann, A., Flicek, P. and Cunningham, F. (2016). The ensembl variant effect predictor. *Genome Biol.* 17, 122. 10.1186/s13059-016-0974-4 27268795 PMC4893825

[BIO062308C22] Miller, S., Janin, J., Lesk, A. M. and Chothia, C. (1987). Interior and surface of monomeric proteins. *J. Mol. Biol.* 196, 641-656. 10.1016/0022-2836(87)90038-6 3681970

[BIO062308C23] Mossey, P. A., Little, J., Munger, R. G., Dixon, M. J. and Shaw, W. C. (2009). Cleft lip and palate. *The Lancet* 374, 1773-1785. 10.1016/S0140-6736(09)60695-4 19747722

[BIO062308C24] Nath, A. S., Parsons, B. D., Makdissi, S., Chilvers, R. L., Mu, Y., Weaver, C. M., Euodia, I., Fitze, K. A., Long, J., Scur, M. et al. (2022). Modulation of the cell membrane lipid milieu by peroxisomal β-oxidation induces Rho1 signaling to trigger inflammatory responses. *Cell Rep.* 38, 110433. 10.1016/j.celrep.2022.110433 35235794

[BIO062308C25] Odrzywolski, A., Tüysüz, B., Debeer, P., Souche, E., Voet, A., Dimitrov, B., Krzesińska, P., Vermeesch, J. R. and Tylzanowski, P. (2024). Gollop–wolfgang complex is associated with a monoallelic variation in WNT11. *Genes (Basel)* 15, 129. 10.3390/genes15010129 38275609 PMC10815061

[BIO062308C26] Pettersen, E. F., Goddard, T. D., Huang, C. C., Meng, E. C., Couch, G. S., Croll, T. I., Morris, J. H. and Ferrin, T. E. (2021). UCSF ChimeraX: structure visualization for researchers, educators, and developers. *Protein Sci.* 30, 70-82. 10.1002/pro.3943 32881101 PMC7737788

[BIO062308C27] Piñero, J., Ramírez-Anguita, J. M., Saüch-Pitarch, J., Ronzano, F., Centeno, E., Sanz, F. and Furlong, L. I. (2020). The DisGeNET knowledge platform for disease genomics: 2019 update. *Nucleic Acids Res.* 48, D845-D855. 10.1093/nar/gkz1021 31680165 PMC7145631

[BIO062308C28] R Core Team (2024). R: A language and environment for statistical computing. R Foundation for Statistical Computing, Vienna, Austria. Available at: https://www.R-project.org/

[BIO062308C29] Richards, S., Aziz, N., Bale, S., Bick, D., Das, S., Gastier-Foster, J., Grody, W. W., Hegde, M., Lyon, E., Spector, E. et al. (2015). Standards and guidelines for the interpretation of sequence variants: a joint consensus recommendation of the American college of medical genetics and genomics and the association for molecular pathology. *Genet. Med.* 17, 405-424. 10.1038/gim.2015.30 25741868 PMC4544753

[BIO062308C30] Robinson, P. N., Köhler, S., Oellrich, A., Genetics, S. M., Wang, K., Mungall, C. J., Washington, N., Bauer, S., Seelow, D., Krawitz, P. et al. (2014). Improved exome prioritization of disease genes through cross-species phenotype comparison. *Genome Res.* 24, 340-348. 10.1101/gr.160325.113 24162188 PMC3912424

[BIO062308C31] Rodrigues, C. H. M., Pires, D. E. V. and Ascher, D. B. (2021). DynaMut2: Assessing changes in stability and flexibility upon single and multiple point missense mutations. *Protein Sci.* 30, 60-69. 10.1002/pro.3942 32881105 PMC7737773

[BIO062308C32] Rooryck, C., Diaz-Font, A., Osborn, D. P. S., Chabchoub, E., Hernandez-Hernandez, V., Shamseldin, H., Kenny, J., Waters, A., Jenkins, D., Kaissi, A. A. et al. (2011). Mutations in lectin complement pathway genes COLEC11 and MASP1 cause 3MC syndrome. *Nat. Genet.* 43, 197-203. 10.1038/ng.757 21258343 PMC3045628

[BIO062308C33] Shapovalov, M. V. and Dunbrack, R. L. (2011). A smoothed backbone-dependent rotamer library for proteins derived from adaptive kernel density estimates and regressions. *Structure* 19, 844-858. 10.1016/j.str.2011.03.019 21645855 PMC3118414

[BIO062308C34] Starbuck, J. M., Ghoneima, A. and Kula, K. (2015). A multivariate analysis of unilateral cleft lip and palate facial skeletal morphology. *J. Craniofac. Surg.* 26, 1673-1678. 10.1097/SCS.0000000000001836 26163844

[BIO062308C35] Steinberg, S. J., Dodt, G., Raymond G, V., Braverman, N. E., Moser, A. B. and Moser, H. W. (2006). Peroxisome biogenesis disorders. *Biochim. Biophys. Acta* 1763, 1733-1748. 10.1016/j.bbamcr.2006.09.010 17055079

[BIO062308C36] Stuppia, L., Capogreco, M., Marzo, G., La Rovere, D., Antonucci, I., Gatta, V., Palka, G., Mortellaro, C. and Tetè, S. (2011). Genetics of syndromic and nonsyndromic cleft lip and palate. *J. Craniofac. Surg.* 22, 1722-1726. 10.1097/SCS.0b013e31822e5e4d 21959420

[BIO062308C37] Walker, M. and Kimmel, C. (2007). A two-color acid-free cartilage and bone stain for zebrafish larvae. *Biotech. Histochem.* 82, 23-28. 10.1080/10520290701333558 17510811

[BIO062308C38] Wang, B. D., Massa, F., Motamedi, F. J., Vidal, V., Rocha, A. S., Gregoire, E. P., Cai, C.-L., Wagner, K. D. and Schedl, A. (2018). Wnt-PEX signaling axis: Connecting developmental pathways with peroxisomal functions in craniofacial morphogenesis. *Dev. Biol.* 441, 42-57. 10.1016/j.ydbio.2018.05.024 29859889 PMC6365680

[BIO062308C39] Warde-Farley, D., Donaldson, S. L., Comes, O., Zuberi, K., Badrawi, R., Chao, P., Franz, M., Grouios, C., Kazi, F., Lopes, C. T. et al. (2010). The GeneMANIA prediction server: biological network integration for gene prioritization and predicting gene function. *Nucleic Acids Res.* 38 Suppl. 2, W214-W220. 10.1093/nar/gkq537 20576703 PMC2896186

[BIO062308C40] Yang, G., Sun, S., He, J., Wang, Y., Ren, T., He, H. and Gao, J. (2023). Enoyl-CoA hydratase/3-hydroxyacyl CoA dehydrogenase is essential for the production of DHA in zebrafish. *J. Lipid Res.* 64, 100326. 10.1016/j.jlr.2022.100326 36592657 PMC9974443

[BIO062308C41] Yu, G. (2024). Thirteen years of clusterProfiler. *Innov.* 5, 100722. 10.1016/j.xinn.2024.100722 PMC11551487 39529960

[BIO062308C42] Zhou, Y., Zhou, B., Pache, L., Chang, M., Khodabakhshi, A. H., Tanaseichuk, O., Schlott, N., Henrichs, K., Sann, H., Trinter, F. et al. (2019). Metascape provides a biologist-oriented resource for the analysis of systems-level datasets. *Nat. Commun.* 10, 1-10. 10.1038/s41467-018-07882-8 30944313 PMC6447622

