# Supplementary material for: Compound inheritance of EHHADH and MASP1 mutations contributes to nonsyndromic cleft lip: familial analysis and zebrafish models
Source: Biol Open. 2025 Dec 11;14(12):bio062308. doi: 10.1242/bio.062308 (PMC12746707; doi:10.1242/bio.062308)
Supplement: Supplementary information [file biolopen-14-062308-s1.pdf]

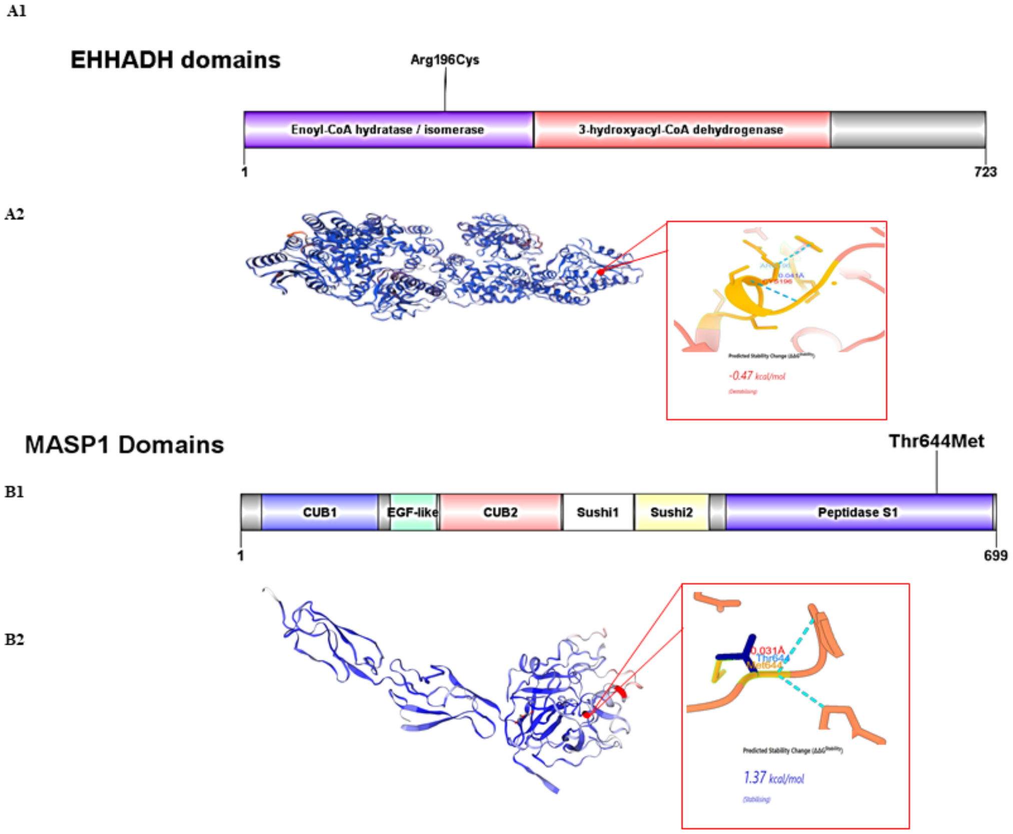

Fig. S1. Domain architecture and structural impact of identified mutations.

(A1) Schematic representation of *EHHADH* protein domains showing the location of the Arg196Cys mutation within the 3-hydroxyacyl-CoA dehydrogenase domain (generated using DOG 1.0: Illustrator of Protein Domain Structures)(Ren J, Wen L, Gao X, Jin C, Xue Y, Yao X. DOG 1.0: illustrator of protein domain structures. *Cell Res.* 2009;19(2):271-3. https://doi.org/10.1038/cr.2009.6). **(A2)** Three-dimensional structure of *EHHADH* protein with detailed view of the R196C mutation site (inset). Structural analysis using UCSF ChimeraX with DynaMut2 stability prediction reveals significant destabilization (ΔΔG = -0.47 kcal/mol). Wild-type protein structure obtained from AlphaFold Database (AF-Q08426-F1-model_v4.pdb). **(B1)** Schematic representation of MASP1 protein domains indicating the position of the Thr644Met mutation within the peptidase S1 domain. **(B2)** Three-dimensional structure of MASP1 protein with detailed view of the T644M mutation site (inset). Structural analysis demonstrates a stabilizing effect (ΔΔG = 1.37 kcal/mol) but with altered local interaction networks. Wild type protein structure obtained from AlphaFold Database (AF-P48740-F1-model_v4.pdb).

Biology Open • Supplementary information

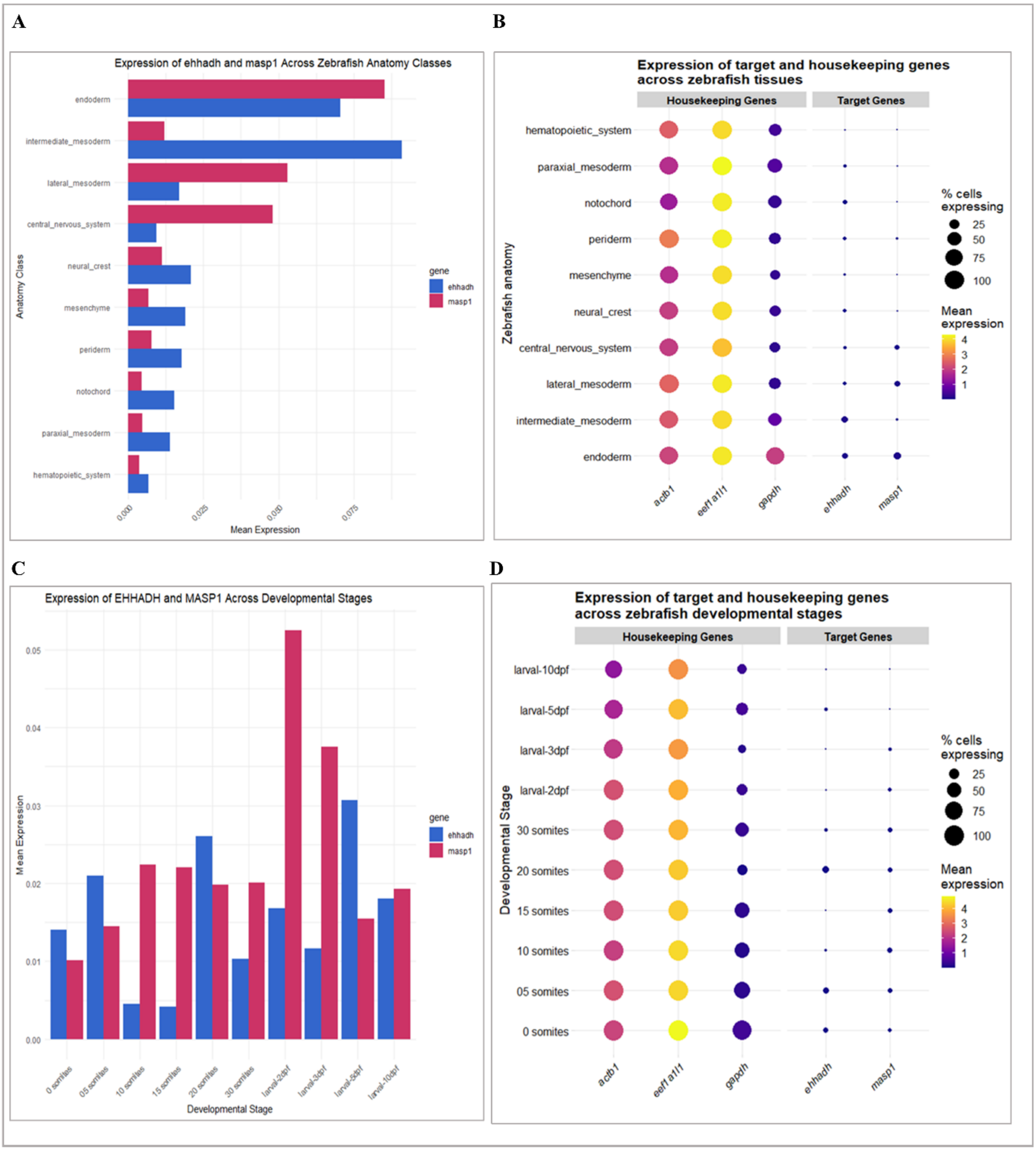

Fig. S2. Expression patterns of *EHHADH* and *MASP1* genes during zebrafish craniofacial development. Single-cell RNA sequencing data from zebrafish development was analyzed using the Zebrafish Hub atlas. Housekeeping genes (*actb1*, *eef1a1l1*, *gapdh*) were included as reference controls. Only developmental stages and anatomical structures with ≥30 cells were analyzed. **(a)** Tissue-specific expression analysis showing mean expression levels across anatomical structures. Dot size represents percentage of cells expressing each gene; color intensity represents mean expression level. *EHHADH* shows highest expression in endoderm (~0.18), moderate expression in intermediate mesoderm (~0.08) and neural crest tissues (~0.02-0.03). *MASP1* displays moderate expression in endoderm (~0.08-0.10), lateral mesoderm (~0.05), and central nervous

Biology Open • Supplementary information

system (~0.08). **(b)** Comparative expression analysis between target genes and housekeeping genes across all tissues, demonstrating restricted expression patterns for target genes compared to ubiquitous housekeeping gene expression. **(c)** Temporal expression analysis across developmental stages. *MASP1* peaks at larval-2dpf during neural crest migration; *EHHADH* peaks at larval-5dpf during cartilage maturation. **(d)** Detailed developmental stage analysis showing mean expression levels (bars) and percentage of expressing cells (dots). The spatiotemporal analysis supports sequential roles: *MASP1* in early neural crest migration and *EHHADH* in later cartilage maturation, providing mechanistic framework for synergistic disruption in compound inheritance.

Table S1. Quantification of phenotypes in zebrafish embryos at 48 hpf

Condition	Total alive	Normal n(%)	Medium n(%)	Strong n(%)	Total abnormal %
GFP	93	92 (98,9)	1 (1,1)	0 (0,0)	1,10
MASP1 WT	135	104 (77,0)	20 (14,8)	11 (8,1)	23
MASP1 MT	146	106 (72,6)	28 (19,2)	12 (8,2)	27,4
EHHADH WT	127	105 (82,7)	14 (11,0)	8 (6,3)	17,3
EHHADH MT	94	68 (72,3)	18 (19,1)	8 (8.5)	27,6
EHHADH+ MASP1 WT	150	118 (78,7)	20 (13,3)	12 (8,0)	21,3
EHHADH+ MASP1 MT	152	100 (65,8)	39 (25,7)	13 (8,6)	34,2

Abbreviations: WT, wild-type; MT, mutant; n, number; hpf, hours post-fertilization

Table S2. Statistical comparisons of phenotypic distributions

Comparison	x^2 (df=1)	p-value	Interpretation
**Individual conditions vs GFP control:**			
GFP vs EHHADH+MASP1 MT	26.26	<0.0001	****
GFP vs EHHADH+MASP1 WT	16.07	0,0003	***
GFP vs MASP1 WT	17.25	0,0002	***
GFP vs MASP1 MT	20.88	<0,0001	****
GFP vs EHHADH WT	12.63	0,0018	**
GFP vs EHHADH MT	20.29	<0,0001	****

Significance levels: ****p<0.0001, ***p<0.001, **p<0.01, *p<0.05, ns=not significant

Biology Open • Supplementary information

Table S3. Ceratohyal angle measurements across experimental conditions

zf ceratohyal angle	GFP	EHHADH WT	EHHAD H MT	masp1 WT	masp1 MT	EHHADH WT+MASP1 WT	EHHADH MT+MASP1 MT
1	74,6	67,2	53,7	51,7	62,7	76,8	122,7
2	75,5	66	48,3	59,7	65,2	87,2	75,6
3	77	50,1	51,2	75,1	53,6	102,8	80
4	71,3	65,2	67,6	74,1	58,3	78,4	82,5
5	76,4	48,2	49,2	67,7	62,4	99,1	85
6	66,6	76	62,6	74,3	52,1	97,9	114,6
7	85,9	55	53,2	53,8	63,2	104,3	62
8	68,6	65,4	49,2	48,9	64,3	79,2	63,7
9	67,6	68,6	51,9	69,4	53,1	94,5	66,7
10	76,1	56,3	65,2	51,1	60,8	86	71,3
11	75,2	49,3	48,2	67,7	57,8	104	77,8
12	73,6	58,3	53,2	66,5	66,3	108,7	85,9
13	68,3	67,6	50,1	65,7	54,8	96,5	87,9
14	74,9	66,2	50	63,8	52,8	89,4	73,2
15	72,8	55	53,2	54,1	57,8	103,2	85,7
16	70	57,3	48	56	52	85	74
17	72,5	58,5	51,5	60,5	56,5	90	78,5
18	73,6	61	53,8	62,9	59	93,9	82,3
19	76	64,2	57,2	67	62,5	98,5	87
20	68,4	58,3	58,4	68,2	65	101,95	89,7
